# A molecular survey on host specificity of feline and canine *Hepatozoon *in model site of northern Kenya

**DOI:** 10.1186/1756-3305-7-S1-O22

**Published:** 2014-04-01

**Authors:** M Gallusová, G Baneth, MA Qablan, AD Mihalca, D Modrý

**Affiliations:** 1 Department of Pathological Morphology and Parasitology, University of Veterinary and Pharmaceutical Sciences Brno, Czech Republic; 2 School of Veterinary Medicine, Hebrew University of Jerusalem, Israel; 3 Institute of Parasitology, Biology Centre, and Faculty of Sciences, University of South Bohemia, České Budějovice, Czech Republic; 4 Department of Parasitology and Parasitic Diseases, University of Agricultural Sciences and Veterinary Medicine, Cluj-Napoca, Romania; 5 CEITEC - Central European Institute of Technology (CZ.1.05/1.1.00/02.0068) from European Regional Development Fund, Brno, Czech Republic

## 

Species of the genus *Hepatozoon *are apicomplexan parasites transmitted by variety of hematophagous arthropods to a wide range of intermediate hosts. *Hepatozoon *infections are broadly distributed around the world and the presence of this parasite is confirmed both in cats and dogs. Until now, two species from canines (*H. canis*, *H. americanum*) and a single species from felines (*H. felis*) have been reported. While the canine *Hepatozoon *spp. are transmitted by ticks, the vector for *H. felis *remains unknown. The aim of presented study was to determine the prevalence and the diversity of *Hepatozoon *in population of domestic cats and dogs living in close contact in rural communities of Samburu pastoralists in northern Kenya and to evaluate the existence of possible cross-transmissions between both hosts. Between years 2007-2012 in total, 135 and 258 blood samples from cats and dogs from the area of Mt. Kulal (5 localities) was collected, respectively. The DNA from blood (preserved in ethanol) was extracted using phenol-chlorophorm method and followed by conventional PCR screening. First round of PCR was done by using Piroplasmid-F and Piroplasmid-R primers, amplifying 18S rRNA gene of *Hepatozoon *spp. (400 bp). A second PCR assay was performed in samples positive by Piroplasmid primers to amplify a larger fragment (1400 bp) of the 18S rRNA gene. All PCR amplicons comming from positive samples were sequenced and determined according to the BLAST match. In cats, this revealed 110 out of 135 (81.5%) positive samples, of which we obtained 104 sequences with an identity of 98% -100% to an existing GenBank accession. In total, 77 sequences were confirmed as *Hepatozoon felis*, 26 samples was proven to be *Hepatozoon *sp. and a single sample was determined as *Hepatozoon canis *with an identity of 96%. Regarding dogs, 121 out of 258 (47%) were positive and we gained 107 sequences of which 105 showed similarity to *Hepatozoon canis *and the remaining two proved to be *Hepatozoon *sp. Despite a close contact of hosts, obviously cats and dogs do not share the same parasite, which demonstrates a great majority of clearly identified *Hepatozoon canis*/*Hepatozoon felis*. Mentioned facts could be explained either by strict host specificity or by presence of different vector or by both statements.

